# Differential contribution of ACh‐muscarinic and *β*‐adrenergic receptors to vasodilatation in noncontracting muscle during voluntary one‐legged exercise

**DOI:** 10.14814/phy2.12202

**Published:** 2014-11-20

**Authors:** Kei Ishii, Kanji Matsukawa, Nan Liang, Kana Endo, Mitsuhiro Idesako, Hironobu Hamada, Tsuyoshi Kataoka, Kazumi Ueno, Tae Watanabe, Makoto Takahashi

**Affiliations:** 1 Department of Integrative Physiology, Graduate School of Biomedical and Health Sciences, Hiroshima University, Hiroshima, Japan; 2 Department of Physical Analysis and Therapeutic Sciences, Graduate School of Biomedical and Health Sciences, Hiroshima University, Hiroshima, Japan; 3 Department of Health Care for Adults, Graduate School of Biomedical and Health Sciences, Hiroshima University, Hiroshima, Japan; 4 Department of Biomechanics, Graduate School of Biomedical and Health Sciences, Hiroshima University, Hiroshima, Japan

**Keywords:** Central command, cholinergic and *β*‐adrenergic vasodilatation, near‐infrared spectroscopy, ultrasound Doppler flowmetry

## Abstract

We have demonstrated the centrally induced cholinergic vasodilatation in skeletal muscle at the early period of voluntary one‐legged exercise and during motor imagery in humans. The purpose of this study was to examine whether central command may also cause *β*‐adrenergic vasodilatation during the exercise and motor imagery. Relative changes in oxygenated hemoglobin concentration (Oxy‐Hb) of bilateral vastus lateralis (VL) muscles, as index of tissue blood flow, and femoral blood flow to nonexercising limb were measured during one‐legged cycling and mental imagery of the exercise for 1 min before and after propranolol (0.1 mg/kg iv). The Oxy‐Hb of noncontracting muscle increased (*P *<**0.05) at the early period of exercise and the increase was sustained throughout exercise, whereas the Oxy‐Hb of contracting muscle increased at the early period but thereafter decreased. We subtracted the Oxy‐Hb response with propranolol from the control response in individual subjects to identify the propranolol‐sensitive component of the Oxy‐Hb response during exercise. In both noncontracting and contracting VL muscles, the increase in Oxy‐Hb at the early period of one‐legged exercise did not involve a significant propranolol‐sensitive component. However, as the exercise proceeded, the propranolol‐sensitive component of the Oxy‐Hb response was developed during the later period of exercise. Propranolol also failed to affect the initial increases in femoral blood flow and vascular conductance of nonexercising leg but significantly attenuated (*P *<**0.05) their later increases during exercise. Subsequent atropine (10–15 *μ*g/kg iv) abolished the initial increases in Oxy‐Hb of both VL muscles. Mental imagery of the one‐legged exercise caused the bilateral increases in Oxy‐Hb, which were not altered by propranolol but abolished by subsequent atropine. It is likely that the rapid cholinergic and delayed *β*‐adrenergic vasodilator mechanisms cooperate to increase muscle blood flow during exercise.

## Abstract (precis)

Oxygenated hemoglobin concentrations (Oxy‐Hb) of noncontracting and contracting vastus lateralis (VL) muscles were monitored with near‐infrared spectroscopy to examine the effects of ACh‐muscarinic and *β*‐adrenergic blockades on the muscular tissue blood flow responses during voluntary one‐legged cycling. In both VL muscles, the propranolol‐sensitive component of the Oxy‐Hb response (ΔOxy‐Hb_PROP_) increased gradually during the later period of the exercise, whereas both atropine‐sensitive component (ΔOxy‐Hb_ATR_) and combined atropine and propranolol‐sensitive component of the Oxy‐Hb response (ΔOxy‐Hb_ATR+PROP_) started to increase from the early period to the end of the exercise. Thus, it is likely that the rapid cholinergic and delayed *β*‐adrenergic vasodilator mechanism operates to increase blood flows of noncontracting and contracting muscles during one‐legged dynamic exercise.

## Introduction

It has been thought that exercise hyperemia results from dilatation of small arterial vessels in contracting muscle by locally released vasoactive substances and mechanical deformation rather than neurally mediated vasodilatation (Saltin et al. 1998; Wray et al. 2005; Clifford 2007; Joyner and Wilkins 2007; Kirby et al. 2007; Golub et al. 2014). In contrast, the vasodilatation observed in the nonexercising limb during voluntary static or dynamic exercise is predominantly mediated by neural and hormonal mechanisms, because muscle contraction is absent in nonexercising limb and no metabolites are released (Duprez et al. 1989; Taylor et al. 1989; Fisher and White 2003; Yoshizawa et al. 2008). The sympathetic cholinergic system is an attractive candidate responsible for the muscle vasodilatation in the nonexercising limb (Abrahams et al. 1960; Shepherd 1983; Sanders et al. 1989; Matsukawa et al. 1997, 2013). We have recently examined this possibility using an estimate of muscle tissue blood flow with near‐infrared spectroscopy (NIRS) in humans (Ishii et al. 2012, 2013). What we found was that atropine abolished the increase in tissue blood flow of the contralateral noncontracting vastus lateralis (VL) muscle at the early period of voluntary one‐legged cycling and that the atropine‐sensitive component of the tissue blood flow response increased rapidly and plateaued in approximately 20 s from the exercise onset (Ishii et al. 2013). Furthermore, atropine abolished vasodilatation in the contralateral VL muscle observed during mental imagery of the one‐legged exercise, which is supposed to simulate central control of the cardiovascular adaptation to exercise without any feedback from contracting muscle (Decety et al. 1993; Williamson et al. 2002; Ishii et al. 2012, 2013). Thus, it is likely that the cholinergic vasodilatation elicited by a central feedforward mechanism (termed central command) plays an important role in the initial muscle vasodilatation of the noncontracting muscle, especially within 20 s from the exercise onset.

On the other hand, *β*‐adrenergic receptors may also contribute to vasodilatation of noncontracting muscle during exercise, because local *β*‐adrenergic blockade attenuated the decrease in vascular resistance of contralateral nonexercising forearm during isometric handgrip (Eklund and Kaijser 1976). *β*‐adrenergic receptors are activated by norepinephrine released from muscle sympathetic nerves and/or epinephrine secreted from the adrenal medulla. However, the responses in muscle sympathetic nerve activity (MSNA) of a resting limb during exercise were not consistent [increased (Herr et al. 1999), decreased (Saito and Mano 1991), and unchanged (Ray et al. 1993)]. Even if MSNA increases, neurally released norepinephrine may cause vasoconstriction according to preferential binding with *α*‐adrenergic receptors. Instead, epinephrine is a more potent agonist on *β*‐adrenergic receptors than *α*‐adrenergic receptors. Activation of adrenal preganglionic sympathetic nerve causes epinephrine secretion (Vissing et al. 1991). When the adrenal preganglionic sympathetic nerve is activated at the exercise onset, secreted epinephrine will reach arterial vessels of skeletal muscle with a time delay of approximately 45 s (Allen et al. 1946; Fukuda et al. 2001; Wakasugi et al. 2010) and thereby may evoke *β*‐adrenergic vasodilatation. On the other hand, if central command activates adrenal preganglionic sympathetic nerve prior to voluntary exercise, feedforward secretion of epinephrine may contribute to the *β*‐adrenergic vasodilatation with a little time delay from the exercise onset (French et al. 2007; Tsuchimochi et al. 2010). It was unclear whether *β*‐adrenergic vasodilatation starts within 20 s or in 45 s from the exercise onset. It was also unknown whether central command is responsible for the *β*‐adrenergic vasodilatation as well as the cholinergic vasodilatation (Ishii et al. 2013). The aim of this study was therefore (1) to examine the effects of *β*‐adrenergic blockade on the tissue blood flow responses of noncontracting and contracting VL muscles with NIRS during voluntary one‐legged cycling; (2) to isolate the ACh (acetylcholine)‐muscarinic and *β*‐adrenergic receptor‐mediated components of the muscle tissue blood flow responses; and (3) to examine whether *β*‐adrenergic vasodilatation is centrally elicited in association with mental imagery of the exercise. A part of this study has been published in a preliminary form (Ishii et al. 2014).

## Methods

### Subjects

Twelve healthy men (age, 23 ± 1 years; height, 173 ±3 cm; body weight, 64 ± 2 kg) participated in this study. None of the subjects suffered from any known cardiovascular and neuromuscular disease. They did not take any medications. The experimental procedures and protocols were performed in accordance with the *Declaration of Helsinki* and approved by the Institutional Ethical Committee. The subjects gave their informed written consent prior to the experiments. All experiments were performed in a soundproof room, in which temperature was maintained 24–26°C.

### One‐legged cycling exercise

Voluntary one‐legged exercise with the right leg was performed for 1 min at 50 rpm in the supine position on a comfortable reclining seat of a specially designed cycle ergometer (*Strength Ergo* 240 BK‐ERG‐003; Mitsubishi Electric Engineering, Tokyo, Japan). The right foot was placed on a shoe affixed at the pedal. The positions of the crank, pedal, and seat were adjusted so that the subjects remained in a comfortable and certain posture. Torque against the wheel shaft and pedal displacement of the ergometer were continuously measured. The subjects were instructed to arbitrarily start one‐legged cycling without any cue and to maintain the left leg relaxed throughout the experiment. On a separate day prior to the main experiment, the subjects were acclimated to the one‐legged exercise in the laboratory environment and performed an incremental one‐legged exercise test to determine the maximal voluntary effort (MVE) as previously reported (Ishii et al. 2012). According to the Borg 6–20 unit scale, the rating of perceived exertion (RPE) was asked after each bout of exercise.

### Mental imagery of one‐legged exercise

To examine the central influence on muscle blood flow without any feedback from contracting muscle, the subjects were instructed to imagine one‐legged cycling of the right leg (cycling imagery) for 1 min as soon as a cue was given (Ishii et al. 2012, 2013). As control, they imagined a circle (drawn on a paper) with no relation to exercise (circle‐imagery). The vividness score of imagery [from 0 (not vivid at all) to 10 (the most vivid)] was asked after each imagery task as previously reported (Williamson et al. 2002; Ishii et al. 2012, 2013).

### Measurements of muscle blood flow

The relative concentrations of oxygenated‐ and deoxygenated hemoglobin (Oxy‐ and Deoxy‐Hb) of the bilateral VL muscles were measured with NIRS. The basic principle of NIRS is that near‐infrared light from three laser photodiodes with different wavelengths penetrates skeletal muscle tissue and some of the light is absorbed by Hb, myoglobin (Mb), and cytochromes and that the remaining light scattered by the tissue is picked up with photodetectors (Boushel and Piantadosi 2000; McCully and Hamaoka 2000). It has been indicated that the Hb in blood vessels of muscle tissue rather than Mb and cytochromes chiefly affects the signals of NIRS (Seiyama et al. 1988). The muscle oxygenation signals of NIRS are dependent on a balance of oxygen supply and utilization in the tissue. As long as oxygen utilization of a muscle remains constant, the signal of Deoxy‐Hb will be constant at a resting level. In this study, the Deoxy‐Hb of the noncontracting VL muscle remained at or near the baseline level throughout the experimental interventions. Also, it was noted that the Deoxy‐Hb of the contracting VL muscle was unchanged at the early period of the one‐legged exercise, suggesting that anaerobically derived ATP seemed to be utilized in the contracting muscle cells and thereby the oxygen utilization might be constant at that period of exercise. In these situations, the signal of Oxy‐Hb was considered as an estimate of muscle tissue blood flow (Ishii et al. 2012, 2013). A pair of photoemission and photodetection probes were placed two‐thirds from the greater trochanter to the top of the patella and attached 4 cm apart on the skin over the left and right VL muscles, so that near‐infrared light intersected the muscle bundles. The reflected near‐infrared light (wavelength: 775 nm, 810 nm, and 850 nm) through muscle tissue was sampled at a rate of 6 Hz and converted to optical densities with a near‐infrared spectrometer (NIRO 200; Hamamatsu Photonics, Hamamatsu, Japan).

### Measurement of femoral blood flow

Blood flow of the left femoral artery was measured with a high‐resolution ultrasound Doppler instrument (Vivid S5; GE Healthcare, Tokyo, Japan) as previously reported (Ishii et al. 2012). A 7.75 MHz linear probe was placed below the inguinal ligament and approximately 2–3 cm above the bifurcation into the profundus and superficial branches. Blood flow velocity measurements were performed with an insonation angle less than 60° and were corrected for the insonation angle. Care was taken to ensure that the probe position and the insonation angle were appropriate and stable and that the sample area was positioned in the center of the vessel and adjusted to cover the vessel caliber. Ultrasound B‐mode images were used to measure systolic and diastolic internal diameter of the femoral artery. A pulsed‐Doppler mode was used to measure femoral blood flow velocity (FBV). The mean femoral internal diameter was calculated based on the following equation (Sato et al. 2011; Ishii et al. 2012): mean internal diameter = [(systolic internal diameter × 1/3)] + [(diastolic internal diameter × 2/3)]. The average values of mean femoral internal diameter and FBV over a period of 5 s were sequentially calculated. At each time period, femoral blood volume flow was calculated by multiplying the cross‐sectional area [*π *× (mean internal diameter/2)^2^] with FBV, and femoral vascular conductance was calculated by dividing femoral blood flow by mean arterial blood pressure (MAP).

### Cardiovascular and electromyogram recordings

An electrocardiogram (ECG) was monitored with a telemetry system (DynaScope DS‐3140; Fukuda Denshi, Tokyo, Japan). Arterial blood pressure (AP) was noninvasively and continuously measured with a Finometer^®^ (Finapres Medical Systems BV, Arnhem, the Netherlands), whose cuff was attached to the left middle finger. The AP waveform was sampled at a frequency of 200 Hz. The beat‐to‐beat values of systolic, diastolic, and MAP and heart rate (HR) were obtained throughout the experiments. Simultaneously, the beat‐to‐beat values of cardiac output (CO), stroke volume (SV), and total peripheral resistance (TPR) were calculated from the aortic pressure waveform using a Modelflow^®^ software (BeatScope 1.1; Finapres Medical Systems BV, Arnhem, the Netherlands). The reliability of the CO measurement using the Modelflow^®^ has been confirmed previously (van Lieshout et al. 2003; Matsukawa et al. 2004; Tam et al. 2004).

Electromyogram (EMG) activity of the VL muscle was bilaterally measured using a pair of silver‐bar electrodes attached on the central portion of the muscle belly (Bagnoli‐2 EMG System, Delsys, Boston, MA). The EMG signals were amplified (×10,000) and passed through a bandpass filter between 20 and 2000 Hz.

### Experimental protocols

The developed torque and pedal displacement of the ergometer, cardiovascular variables, and EMG signals of the bilateral VL muscles were simultaneously measured throughout the experiments. We confirmed that the EMG activity of the noncontracting muscle was absent in all conditions. The following protocols were conducted on separate days.

#### Protocol 1: effects of autonomic blockades on Oxy‐Hb responses

With measurement of NIRS signals of the bilateral VL muscles, ten of the 12 subjects performed voluntary one‐legged cycling with 35% of the MVE, cycling imagery, and circle‐imagery in the absence of any drug (control condition). Then propranolol hydrochloride (0.1 mg/kg) was intravenously administrated into the right cephalic vein. After a rest period of 6 ± 0.2 min from the injection, each individual task was conducted under *β*‐adrenergic blockade (propranolol condition). Subsequently, atropine sulfate (10–15 *μ*g/kg) and propranolol (one‐third of the initial dose) were injected. After a rest period of 7 ± 0.3 min from the subsequent injection, each individual task was conducted again under both muscarinic and *β*‐adrenergic blockades (atropine and propranolol condition).

Both atropine and propranolol have the same half‐life period of approximately 4 h (Brown and Taylor 2001; Hoffman 2001). We observed that intravenous administration of propranolol alone decreased baseline HR, whereas combined atropine and propranolol caused an increase in baseline HR, pupil dilatation, and thirsting. These systemic effects continued throughout each experimental condition (i.e., for more than 30 min).

#### Protocol 2: effects of propranolol on blood flow responses

Femoral blood flow to the nonexercising limb during one‐legged cycling was measured before and after propranolol in five of the 12 subjects (three subjects participated in Protocol 1 and newly recruited two subjects). The exercise intensity was reduced to 20% MVE, to minimize movement artifact in the blood flow measurement. Subsequently, atropine and propranolol were injected and femoral blood flow was measured in four of the five subjects.

### Data analysis

The data of the developed torque and pedal displacement of the ergometer, AP, ECG, EMG, and NIRS signals were stored to a computer at a sampling frequency of 1000 Hz (MP150; BIOPACK Systems, Santa Barbara, CA) for off‐line analysis. The motor performance during one‐legged exercise was unaffected by any drugs. The onset of voluntary one‐legged cycling was defined as “time = 0” according to the onset of pedal displacement. The absolute concentration of Oxy‐Hb could not be obtained, because the pathlength of near‐infrared light within tissue was unknown *in vivo*. Instead, the relative changes in Oxy‐Hb were expressed as a percentage against the baseline. The zero level of the Oxy‐Hb was defined as the minimum value of Oxy‐Hb obtained during inflation of a pneumatic cuff, wrapped around the upper thigh, with a pressure of 200–250 mmHg. The baseline value of the Oxy‐Hb signal against the minimum value was taken as 100%. To cancel phasic changes in the NIRS signals due to movement artifact, the NIRS signals of the contracting VL muscle were recalculated using a moving average over neighboring 1000 points.

We subtracted the Oxy‐Hb response with propranolol (termed ΔPROP‐Oxy‐Hb) from the control response (termed ΔCON‐Oxy‐Hb) in individual subjects to identify the propranolol‐sensitive component of the Oxy‐Hb response during exercise [termed ΔOxy‐Hb_PROP_ = (ΔCON‐Oxy‐Hb) − (ΔPROP‐Oxy‐Hb)]. Similarly, the Oxy‐Hb response with both atropine and propranolol (termed ΔATR+PROP‐Oxy‐Hb) was subtracted from the ΔCON‐Oxy‐Hb to determine the atropine and propranolol‐sensitive component of the Oxy‐Hb response [termed ΔOxy‐Hb_ATR+PROP_ = (ΔCON‐Oxy‐Hb) − (ΔATR+PROP‐Oxy‐Hb)]. The changes in Oxy‐ and Deoxy‐Hb, ΔOxy‐Hb_PROP_, ΔOxy‐Hb_ATR+PROP_, HR, and MAP were sequentially averaged every 1 s. The initial changes in the NIRS and Doppler variables during exercise were determined as the peak values at the early period of exercise (5–20 s from the exercise onset). The initial changes in HR and MAP were determined as the values at the time when the Oxy‐Hb of noncontracting muscle reached the initial peak. The later changes in the NIRS and Doppler variables, HR, and MAP were obtained as the averages during the later period (45–60 s) of exercise. In the imagery interventions, the response of each variable was obtained as an average over a time period from 30 to 45 s after the imagery onset as previously reported (Ishii et al. 2012, 2013).

### Statistical analysis

The baseline and peak absolute values of the hemodynamics and Doppler variables during one‐legged exercise were compared among the three conditions (control vs. propranolol vs. atropine and propranolol) by a one‐way ANOVA with repeated measures and a Tukey *post hoc* test. The relative changes in all variables were analyzed using a two‐way ANOVA (2 × 2 or 2 × 3 factorial design) with repeated measures and a Tukey *post hoc* test. The differences in the Oxy‐Hb responses between bilateral VL muscles were also analyzed by a paired *t*‐test. The comparison of the Borg scales and the vividness scores among the conditions was conducted using a Friedman repeated measures ANOVA on ranks with a Tukey *post hoc* test. A level of statistical significance was defined at *P *<**0.05 in all cases. All statistical analyses were performed using SigmaPlot^®^ version 12.5 (Systat Software, San Jose, CA). All variables are expressed as means ± SE.

## Results

### Effects of autonomic blockades on the cardiovascular responses during one‐legged cycling

The effects of propranolol on the baseline hemodynamics are summarized in Table 1. Propranolol significantly (*P *<**0.05) decreased baseline HR, SV, and CO and increased TPR. Following subsequent combined injection of atropine and propranolol, baseline HR increased and baseline SV decreased, whereas baseline CO and TPR returned to the control levels. Baseline MAP was not significantly (*P *>**0.05) affected by either propranolol or subsequent atropine and propranolol as compared to the control, although a slight difference existed (*P *<**0.05) between the two conditions (propranolol vs. atropine and propranolol).

**Table 1.** The effects of propranolol and additional atropine on the baseline and peak changes in hemodynamics and the rating of perceived exertion during one‐legged exercise at 35% of maximal voluntary effort in 10 subjects

	Control			Propranolol			Atropine and propranolol		
	Baseline	During	Change	Baseline	During	Change	Baseline	During	Change
HR (beats/min)	63 ± 2	103 ± 2*	41 ± 1	55 ± 2†	90 ± 2*†	35 ± 1†	74 ± 2†‡	97 ± 2*†‡	23 ± 2†‡
SV (mL)	96 ± 5	108 ± 6*	13 ± 2	91 ± 4†	104 ± 4*	13 ± 2	82 ± 4†‡	99 ± 5*†	17 ± 3
CO (L/min)	6.0 ± 0.2	10.7 ± 0.4*	4.9 ± 0.3	5.0 ± 0.2†	9.0 ± 0.3*†	4.0 ± 0.3†	6.1 ± 0.4‡	9.3 ± 0.4*†	3.2 ± 0.3†‡
MAP (mmHg)	95 ± 2	117 ± 2*	24 ± 1	93 ± 2	110 ± 2*†	17 ± 2†	99 ± 2‡	111 ± 2*†	12 ± 1†‡
TPR (mmHg/L/min)	16 ± 1	10 ± 0.4*	−6 ± 1	19 ± 1†	12 ± 1*†	−8 ± 1†	17 ± 1‡	11 ± 1*†	−5 ± 1‡
RPE (Borg scale)	12.0 ± 0.2			12.6 ± 0.3			13.1 ± 0.2†		

Values are means ± SE. HR, heart rate; SV, stroke volume; CO, cardiac output; MAP, mean arterial blood pressure; TPR, total peripheral resistance; RPE, rating of perceived exertion.

* Significant difference (*P *<**0.05) from the baseline value.

† Significant difference (*P *<**0.05) from the value in the control condition.

‡ Significant difference (*P *<**0.05) between the conditions (propranolol vs. atropine and propranolol).

The peak cardiovascular responses during voluntary one‐legged cycling at 35% MVE are summarized in Table 1. Without any drugs, HR and CO increased 63–78% during the exercise, SV 12%, and MAP 23%, respectively, whereas TPR decreased 38%. Propranolol significantly blunted the peak increases in HR and CO and augmented the peak decrease in TPR, resulting in the blunted response of MAP. Subsequent atropine and propranolol further attenuated (*P *<**0.05) the peak increases in HR, CO, and MAP, but recovered the peak decrease in TPR to the control level. The peak increase in SV was unchanged by any drugs. RPE was not influenced by propranolol but slightly increased (*P *<**0.05) after subsequent atropine and propranolol (Table 1).

### Oxy‐Hb response at the early period of one‐legged cycling

**Figure 1.**
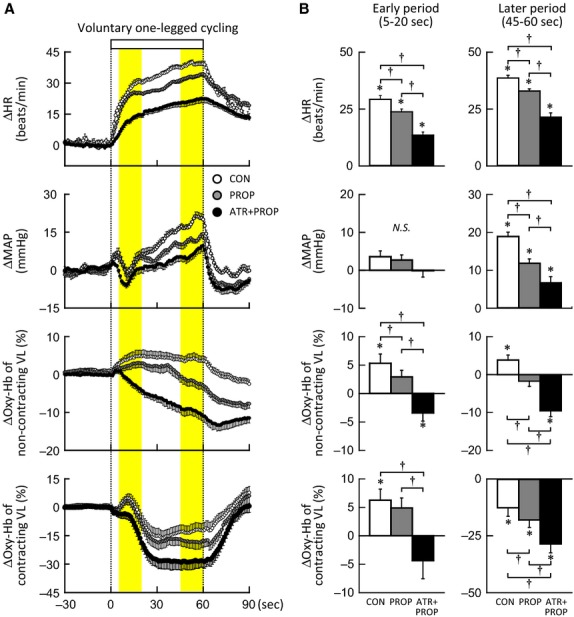
(A) The time courses of the changes in heart rate (HR), and mean arterial blood pressure (MAP), and signal of oxygenated hemoglobin (Oxy‐Hb) of noncontracting and contracting vastus lateralis (VL) muscle during voluntary one‐legged cycling at 35% of maximal voluntary effort (MVE) in the control (○, CON), propranolol (, PROP), and atropine and propranolol (●, ATR+PROP) conditions (*n* = 10 subjects). The relative percent changes in Oxy‐Hb were determined by identifying the zero level with muscle ischemia. Each variable was sequentially calculated every 1 s. Vertical dotted lines indicate the onset and end of one‐legged exercise. Yellow areas indicate the early (5–20 s) and later period (45–60 s) of the exercise. A square bar indicates the duration of exercise. (B) The initial and later changes in HR, MAP, and Oxy‐Hb of noncontracting and contracting VL muscle during one‐legged cycling in the CON (□), PROP () and ATR+PROP (■) (*n* = 10 subjects). *Significant difference (*P *<**0.05) from the baseline. ^†^Significant difference (*P *<**0.05) among the conditions (CON vs. PROP vs. ATR+PROP). *N.S*., not significant.

The Oxy‐Hb of each of noncontracting and contracting VL muscle increased at the early period (5–20 s) of one‐legged cycling without any drugs as shown in Fig. 1A. It is noted that the initial increases in the Oxy‐Hb appeared simultaneously with tachycardia but were unrelated to the MAP response and that they tended to persist after propranolol but disappeared following subsequent atropine and propranolol. Figure 1B summarizes the effects of propranolol on the initial responses in HR, MAP, and the Oxy‐Hb of the noncontracting and contracting VL muscles. The initial increase in HR was blunted (*P *<**0.05) by propranolol and further by subsequent atropine and propranolol. A slight rise in MAP was observed in the control and propranolol conditions, although the MAP changes were not statistically significant (*P *>**0.05). The initial increase of 5 ± 1% in Oxy‐Hb of the noncontracting muscle was slightly attenuated by propranolol (*P *<**0.05). The initial increase in Oxy‐Hb was abolished by subsequent atropine and propranolol and turned out to be a decreased response. In the contracting muscle, the initial increase in Oxy‐Hb was not affected by propranolol but abolished by subsequent atropine and propranolol. On the whole, the initial Oxy‐Hb of both muscles tended to respond similarly in all conditions.

### Oxy‐Hb response during the later period of one‐legged cycling

During the later period (45–60 s) of one‐legged cycling without any drugs, the increase in HR plateaued and the rise in MAP continued (Fig. 1A). The increase in Oxy‐Hb of the noncontracting VL muscle was maintained, whereas the Oxy‐Hb of the contracting VL muscle decreased in progress of oxygen utilization with muscular activity as previously reported (Ishii et al. 2012, 2013). The Oxy‐Hb of both muscles during the later period of exercise was decreased by propranolol and further by subsequent atropine and propranolol. Figure 1B summarizes the effects of propranolol on the later responses in HR, MAP, and the Oxy‐Hb of the noncontracting and contracting muscles. The increases in HR and MAP during the later period of exercise were blunted (*P *<**0.05) by propranolol and further by subsequent atropine and propranolol. The later increase in Oxy‐Hb of the noncontracting muscle was reversed by propranolol (*P *<**0.05) and augmented by subsequent atropine and propranolol. The autonomic blockades had the same effects on the later Oxy‐Hb response between the noncontracting and contracting muscles.

### Drug‐sensitive components of Oxy‐Hb responses during one‐legged cycling

**Figure 2.**
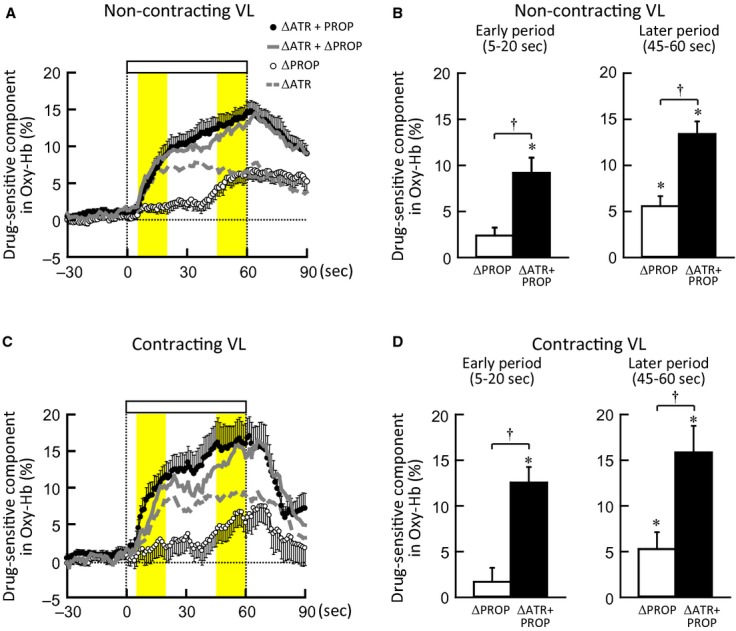
(A and C) The time courses of the ΔOxy‐Hb_PROP_ and ΔOxy‐Hb_ATR__+__PROP_ (taken as the propranolol‐ (○) or atropine and propranolol‐ (●) sensitive component of the Oxy‐Hb responses) of noncontracting (A) and contracting VL muscle (C) during voluntary one‐legged cycling at 35% MVE (*n* = 10 subjects). Dashed gray lines indicate the ΔOxy‐Hb_ATR_ (taken as the atropine‐sensitive component of the Oxy‐Hb responses) obtained from Ishii et al. (2013). Solid gray lines indicate the summation of ΔOxy‐Hb_PROP_ and ΔOxy‐Hb_ATR_. (B and D) The initial and later changes in ΔOxy‐Hb_PROP_ (□) and ΔOxy‐Hb_ATR__+__PROP_ (■) of noncontracting (B) and contracting VL muscle (D) during one‐legged cycling (*n* = 10 subjects). *Significant difference (*P *<**0.05) from the baseline. ^†^Significant difference (*P *<**0.05) between ΔOxy‐Hb_PROP_ and ΔOxy‐Hb_ATR__+__PROP_.

The time courses of the ΔOxy‐Hb_PROP_ (defined as the propranolol‐sensitive component) and ΔOxy‐Hb_ATR+PROP_ (defined as the atropine and propranolol‐sensitive component) during one‐legged cycling are shown in Fig. 2. The ΔOxy‐Hb_PROP_ of the noncontracting VL muscle increased gradually during the later period of the exercise, whereas the ΔOxy‐Hb_ATR+PROP_ of the muscle started to increase from the early period of the exercise and continued to increase until the end of the exercise (Fig. 2A). In the contracting VL muscle (Fig. 2C), the ΔOxy‐Hb_PROP_ and ΔOxy‐Hb_ATR+PROP_ also increased with the similar time courses and magnitudes as those observed in the noncontracting muscle. It was of interest that the ΔOxy‐Hb_ATR+PROP_ response matched an algebraic summation of ΔOxy‐Hb_PROP_ and ΔOxy‐Hb_ATR_ [= (ΔCON‐Oxy‐Hb) − (ΔATR‐Oxy‐Hb), cited from Ishii et al. (2013)] in either VL muscle (Fig. 2A and C). Figure 2B and D summarize the initial and later changes in the ΔOxy‐Hb_PROP_ and ΔOxy‐Hb_ATR+PROP_ of the noncontracting and contracting muscles. The initial and later responses in the ΔOxy‐Hb_PROP_ were not different (*P *>**0.05) between the noncontracting and contracting muscles: the ΔOxy‐Hb_PROP_ did not change significantly (*P *>**0.05) from the baseline at the early period of exercise, whereas the ΔOxy‐Hb_PROP_ increased by 5–6% (*P *<**0.05) during the later period. The initial and later increases in the ΔOxy‐Hb_ATR+PROP_ were much greater (*P *<**0.05) than the ΔOxy‐Hb_PROP_ responses and were identical between both muscles (Fig. 2B and D).

### Femoral blood flow responses to one‐legged cycling before and after propranolol

Following propranolol, baseline FBV, femoral blood flow, and femoral vascular conductance tended to decrease (13 ± 4 vs. 6 ± 2 cm/s, 517 ± 184 vs. 220 ± 86 mL/min, and 5.3 ± 1.8 vs. 2.2 ± 0.9 mL/min/mmHg, respectively) with no changes in mean internal diameter of the femoral artery (0.88 ± 0.04 vs. 0.89 ± 0.05 cm). The decreases were not significant probably due to a small sample size and large intersubject variability.

**Figure 3.**
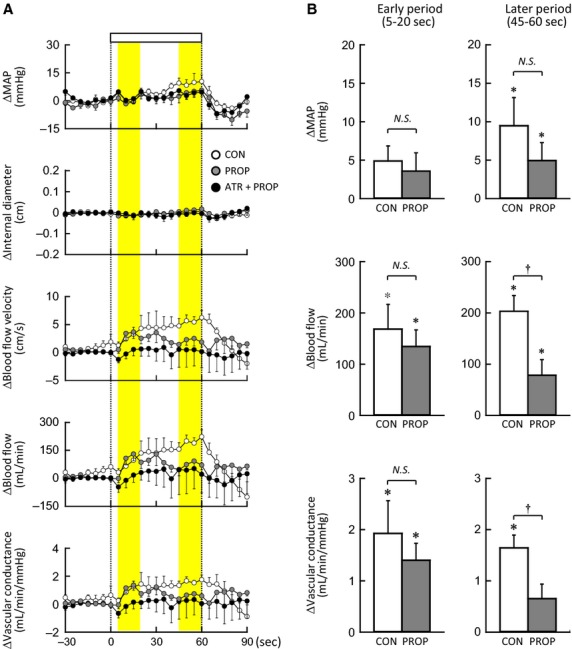
(A) The time courses of the changes in MAP and mean internal diameter, blood flow velocity, blood flow, and vascular conductance of the femoral artery of nonexercising limb during voluntary one‐legged cycling at 20% MVE in the control (○, *n* = 5 subjects), propranolol (, *n* = 5 subjects), and atropine and propranolol conditions (●, *n* = 4 subjects). Each variable was sequentially calculated every 5 s. (B) The initial and later changes in MAP, femoral blood flow, and femoral vascular conductance during voluntary one‐legged cycling at 20% MVE in the control (□) and propranolol conditions () (*n* = 5 subjects). The femoral blood flow data in the atropine and propranolol condition were not statistically analyzed due to the small sample size. CON, control condition. PROP, propranolol condition. ATR+PROP, atropine and propranolol condition. *Significant difference (*P *<**0.05) from the baseline. ^†^Significant difference (*P *<**0.05) between the control and propranolol conditions. *N.S*., not significant (*P *>**0.05).

Figure 3A represents the time courses of the average changes in MAP and the Doppler variables of the nonexercising limb during one‐legged cycling at 20% MVE. Femoral blood flow and vascular conductance as well as FBV increased during the exercise with a gradual rise in MAP, whereas mean internal diameter of the artery was unchanged throughout the exercise. Propranolol hardly affected the initial femoral blood flow responses but blunted their later increases without changing the femoral internal diameter (Fig. 3A). The effects of propranolol on the initial and later changes in MAP, femoral blood flow, and femoral vascular conductance are summarized in Fig. 3B. MAP did not change significantly at the early period but increased during the later period of exercise. The initial and later changes in MAP were not different (*P *>**0.05) between the control and propranolol conditions (Fig. 3B). Although propranolol showed a tendency to attenuate the initial increases in femoral blood flow and vascular conductance, the effects were not significant (*P *>**0.05). In contrast, the later increases in femoral blood flow and vascular conductance were substantially attenuated (*P *<**0.05) by propranolol in Fig. 3B. Subsequent atropine and propranolol tended to abolish the initial increases in femoral blood flow and vascular conductance during the exercise (Fig. 3A).

### The Oxy‐Hb and cardiovascular responses to mental imagery of the one‐legged cycling

**Figure 4.**
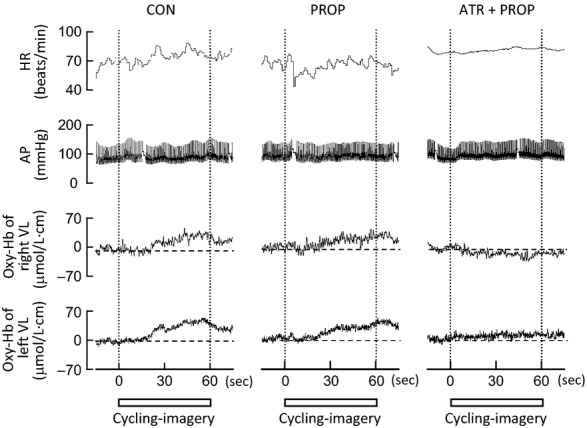
Representative recordings of HR, arterial blood pressure (AP), and Oxy‐Hb of the right and left VL muscles during mental imagery of one‐legged cycling (cycling imagery) under the control (CON), propranolol (PROP), and atropine and propranolol (ATR+PROP) conditions in a subject. Vertical dotted lines indicate the onset and end of the cycling imagery. Horizontal dashed lines indicate the baseline levels of the Oxy‐Hb. Square bars indicate the duration of imagery.

**Figure 5.**
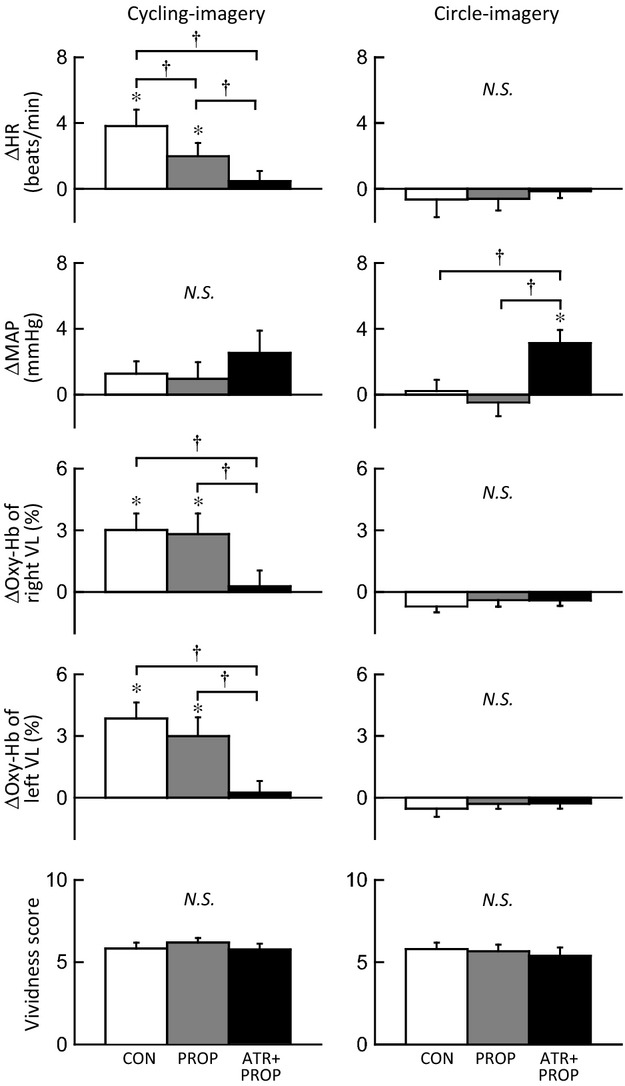
The effects of propranolol and subsequent atropine on the changes in HR, MAP, and Oxy‐Hb of bilateral VL muscles and the vividness score during cycling‐ and circle‐imagery (*n* = 10 subjects). CON, control condition (□). PROP, propranolol condition (). ATR+PROP, atropine and propranolol condition (■). *Significant difference (*P *<**0.05) from the baseline. ^†^Significant difference (*P *<**0.05) among the three conditions (CON vs. PROP vs. ATR+PROP). *N.S*., not significant (*P *>**0.05).

Figure 4 exemplifies typical responses of HR, AP, and the Oxy‐Hb of the right and left VL muscles during mental imagery of the one‐legged cycling in a subject. Cycling imagery induced bilateral increases in Oxy‐Hb without changing AP as previously reported (Ishii et al. 2012, 2013). The increases in Oxy‐Hb were not affected by propranolol but abolished by subsequent atropine and propranolol. Figure 5 summarizes the effects of propranolol and subsequent atropine and propranolol on the imagery‐induced cardiovascular and Oxy‐Hb responses. Slight tachycardia observed during cycling imagery was blunted (*P *<**0.05) by propranolol and abolished by subsequent atropine and propranolol. The increases in Oxy‐Hb of the bilateral muscles during cycling imagery were not significantly affected by propranolol but abolished by subsequent atropine and propranolol. MAP did not change significantly from the baseline level during the cycling imagery in all conditions. On the other hand, circle‐imagery did not cause any rises in HR and the Oxy‐Hb in bilateral VL muscles irrespective of the presence or absence of the drugs, although a slight rise in MAP was recognized following subsequent atropine and propranolol. The vividness score was not different between the two kinds of imagery and was the same irrespective of the presence or absence of the drugs (Fig. 5).

## Discussion

The effects of ACh‐muscarinic and *β*‐adrenergic blockades on the muscular tissue blood flow responses during voluntary one‐legged cycling and mental imagery of the exercise have been examined for the first time in humans. We measured the Oxy‐Hb as an index of muscle tissue blood flow with NIRS. To identify a propranolol‐ or atropine and propranolol‐sensitive component of the Oxy‐Hb response during the exercise, the Oxy‐Hb response under ACh‐muscarinic and/or *β*‐adrenergic blockades was subtracted from the control response. Our major new findings are that (1) in both noncontracting and contracting VL muscles, the increase in Oxy‐Hb at the early period of one‐legged exercise did not involve a significant propranolol‐sensitive component; (2) as the exercise proceeded, the propranolol‐sensitive component of the Oxy‐Hb response developed during the later period of exercise; (3) the atropine and propranolol‐sensitive component of the Oxy‐Hb response increased from the early period to the end of exercise; (4) furthermore, propranolol did not affect the initial increases in femoral blood flow and vascular conductance of the nonexercising leg but attenuated their later increases; (5) mental imagery of the one‐legged cycling caused the bilateral increases in Oxy‐Hb, which were not altered by propranolol but abolished by subsequent atropine. Taken together, it is likely that the centrally induced cholinergic vasodilator mechanism starts to operate from the onset of exercise, whereas the *β*‐adrenergic vasodilator mechanism operates predominantly during the later period of exercise.

### *β*‐adrenergic vasodilatation in noncontracting and contracting muscle during exercise

Regarding noncontracting muscle, Eklund and Kaijser (1976) found that propranolol, but not phentolamine, attenuated a decrease in vascular resistance of contralateral nonexercising forearm during static handgrip, suggesting a *β*‐adrenergic receptor‐mediated vasodilatation. Such vasodilatation may not be caused by norepinephrine released from muscle sympathetic nerves, because of the following reasons. First, the response in MSNA of a resting limb during exercise varied among previous studies [increased (Herr et al. 1999), decreased (Saito and Mano 1991), and unchanged (Ray et al. 1993)]. Second, even if MSNA increases during exercise, neurally released norepinephrine would function chiefly as a vasoconstrictor agent due to a higher potency on *α*‐adrenergic receptors than *β*‐adrenergic receptors. Instead, epinephrine secreted by activation of adrenal preganglionic sympathetic nerve is a more potent agonist on *β*‐adrenergic receptors. Tsuchimochi et al. (2010) found that electrical stimulation of the hypothalamic locomotor region increased adrenal preganglionic sympathetic nerve activity and catecholamine output from the adrenal medulla in anesthetized rats. Under local blockade of unilateral stellate ganglion, mental stress evoked *β*‐adrenergic forearm vasodilatation in humans (Halliwill et al. 1997). It is possible that central descending signal related to exercise may trigger epinephrine secretion. If adrenal preganglionic sympathetic nerve is activated by central command prior to the exercise onset, feedforward secretion of epinephrine should cause *β*‐adrenergic vasodilatation with a little time delay from the exercise onset (French et al. 2007; Tsuchimochi et al. 2010). As opposed to this hypothesis, the Oxy‐Hb response of the noncontracting muscle at the early period of one‐legged cycling contained no significant propranolol‐sensitive component, although the initial Oxy‐Hb response was slightly blunted by propranolol (Figs. 1 and 2). The finding suggests that the *β*‐adrenergic vasodilatation, if any, is not sufficient for evoking the initial increase in blood flow of the noncontracting muscle. This is supported by the upstream femoral blood flow responses (Fig. 3). In contrast, as the exercise proceeded, the propranolol‐sensitive increases in the Oxy‐Hb and femoral vascular conductance became evident, suggesting that the *β*‐adrenergic vasodilatation plays a dominant role in increasing muscle tissue and limb blood flow during the later period of exercise.

In contracting muscle, the propranolol‐sensitive component of the Oxy‐Hb response also appeared during the later period of the exercise (Fig. 2). This finding is in agreement with the previous studies demonstrating that *β*‐adrenergic blockade reduced blood flow to the exercising limb at 1 min after the onset of mild running exercise in rats (Overton 1993) and during graded bicycle exercise at mild to moderate intensity in humans (Gullestad et al. 1993). Laughlin and Armstrong (1987) found using a radiolabeled microsphere technique that propranolol reduced the rise in blood flows to eight of 32 hindlimb muscle samples at 30 s of mild running exercise in rats. They also noticed that the effect of propranolol disappeared at a higher intensity of exercise. Similarly, *β*‐adrenergic blockade failed to reduce blood flow to the exercising limb during running at a moderate intensity in dogs (Buckwalter et al. 1997) and during bicycle exercise at 50% of the maximal oxygen capacity in humans (Juhlin‐Dannfelt and Aström 1979). Taken together, the delayed contribution of *β*‐adrenergic vasodilatation to exercise hyperemia seems apparent during exercise at a mild to moderate intensity but it may be masked by vasodilatation due to contraction‐related factors (such as metabolites) during heavier exercise. Interestingly, it appears that a latent time of the *β*‐adrenergic vasodilatation corresponded to the transit time (approximately 45 s) of epinephrine from the adrenal medulla to systemic arterial vessels (Allen et al. 1946; Fukuda et al. 2001; Wakasugi et al. 2010). Although plasma epinephrine concentration increased at 3–5 min of mild static or dynamic exercise in humans (Gullestad et al. 1993; Pawelczyk et al. 1997; Trombetta et al. 2005), to what extent plasma epinephrine increases within 1 min of the one‐legged exercise remains to be studied.

### A mechanism responsible for *β*‐adrenergic vasodilatation during the later period of exercise

Propranolol had no impact on the increase in Oxy‐Hb during mental imagery of one‐legged exercise (Figs. 4 and 5), suggesting that the central descending signal related to exercise did not evoke *β*‐adrenergic muscle vasodilatation. Instead, *β*‐adrenergic vasodilatation during the later period of exercise may be caused by another mechanism (Vissing et al. 1991): a reflex originated from metabo‐ and/or mechanosensitive afferents in contracting muscles (termed exercise pressor reflex). Activation of metabosensitive afferents by postexercise ischemia failed to increase plasma epinephrine in humans (Pawelczyk et al. 1997) and mechanical stretch of the hindlimb triceps surae muscle increased plasma epinephrine to the same extent as muscle contraction in anesthetized cats (Matsukawa et al. 2001), suggesting that a muscle mechanoreflex, rather than a metaboreflex, causes epinephrine secretion. To assess the effect of the muscle mechanoreflex on the Oxy‐Hb response during one‐legged cycling, passive cycling driven by a motor was conducted in our previous study (Ishii et al. 2012). The Oxy‐Hb of the contralateral VL muscle during the passive one‐legged cycling increased with the same time course as the *β*‐adrenergic component observed during voluntary one‐legged cycling (Fig. 2). Furthermore, the increase in Oxy‐Hb during passive cycling tended to be blunted by propranolol in preliminary experiments (unpublished observation). Thus, it is suggested that a muscle mechanoreflex may contribute to the *β*‐adrenergic vasodilatation during the later period of exercise.

### Summation of cholinergic and *β*‐adrenergic vasodilatation

Whether a sympathetic cholinergic vasodilator mechanism exists in human skeletal muscle is a controversial issue. Previous studies demonstrated using ultrasound Doppler flowmetry that muscarinic blockade had no impact on the increased blood flow to an exercising limb (Shoemaker et al. 1997; Brock et al. 1998). However, since atropine‐sensitive vasodilatation in a resting limb is elicited by handgrip exercise (Sanders et al. 1989) or mental stress (Dietz et al. 1994), it cannot be neglected that sympathetic cholinergic vasodilatation may be masked by the vasodilatation derived metabolically and mechanically in contracting muscle. Along this line, we have recently re‐examined the contribution of sympathetic cholinergic vasodilatation to a rise in muscle blood flow during one‐legged exercise using NIRS (Ishii et al. 2012, 2013). NIRS can estimate tissue blood flow response in a localized region of skeletal muscle at a higher time resolution as compared to a conventional flowmetry. Our previous (Ishii et al. 2013) and present study showed that atropine abolished the increases in Oxy‐Hb of bilateral VL muscles at the early period of one‐legged exercise and during mental imagery of the exercise, suggesting that central command elicits sympathetic cholinergic vasodilatation in noncontracting and contracting muscle.

It is known that the cholinergic neurotransmission is modified by pre‐ and/or post–junctional *β*‐adrenergic receptors in the cardiac atrium, trachea, and ileum, (Furukawa and Levy 1984; Reddy et al. 1995; Zhang et al. 1995). If this is true in skeletal muscle, the cholinergic vasodilatation would be augmented or attenuated under pretreatment of propranolol. However, in both noncontracting and contracting muscles, the atropine and propranolol‐sensitive component of the Oxy‐Hb response (ΔOxy‐Hb_ATR+PROP_) equaled the algebraic summation of the atropine‐sensitive component (ΔOxy‐Hb_ATR_) and propranolol‐sensitive component (ΔOxy‐Hb_PROP_) (Fig. 2). This finding indicates that the two vasodilator mechanisms via muscarinic receptors and *β*‐adrenergic receptors may cooperate in a summative manner during exercise.

### Role of *α*‐adrenergic receptors in muscle blood flow regulation

The double blockade of *β*‐adrenergic and muscarinic receptors disclosed a role of the remaining *α*‐adrenergic receptors in regulation of muscle blood flow. Following subsequent atropine and propranolol, the Oxy‐Hb of noncontracting muscle began to decrease at the early period of the exercise (Fig. 1), suggesting that *α*‐adrenergic vasoconstriction developed from the early period of exercise. Indeed, Herr et al. (1999) and Cui et al. (2006) showed an increase in MSNA within 4–6 s from the onset of thigh or calf muscle contraction using a signal averaging technique. Hansen et al. (1994) observed bilateral increases in MSNA to both nonexercising and exercising limbs during the second minute of toe extension exercise. Generally, it is known that MSNA increases dependently on the exercise intensity and duration (Seals 1989). Taken together, it is likely that a sympathetic *α*‐adrenergic vasoconstrictor signal may be transmitted to noncontracting and contracting muscle from the early period of one‐legged exercise and may become stronger depending on time and intensity of exercise.

### Limitations

The evaluation of blood flow with NIRS and Doppler flowmetry involves some assumptions and limitations. First, NIRS does not directly measure blood flow but provides information for a balance of oxygen supply and utilization in the microcirculation within the illuminated tissue (Mancini et al. 1994; Barrett and Rattigan 2012). As long as oxygen utilization of a muscle remains constant, a relative change in Oxy‐Hb is assumed as an index of muscle tissue blood flow (Ishii et al. 2012, 2013). Second, signals of NIRS are influenced not only by muscle blood flow but by skin blood flow. We have already reported that no changes in skin blood flow were observed during the one‐legged exercise (Ishii et al. 2012), although skin blood flow was not measured in this study. This is also supported by the finding that skin vascular conductance of the forearm or leg was unchanged during isomeric exercise (Saad et al. 2001). Third, the intensity of the one‐legged cycling in the NIRS measurement (Protocol 1) was set at 35% MVE, whereas the exercise intensity was reduced to 20% MVE in the Doppler measurement (Protocol 2) so as to minimize movement artifact. It cannot be denied that the femoral blood flow response might be underestimated compared with the Oxy‐Hb data. Forth, the sample size of the femoral blood flow protocol was low. Nevertheless, femoral blood flow and vascular conductance increased during the exercise with the similar time course and magnitude as those observed in our previous study (Ishii et al. 2012). Furthermore, the raw blood flow data in all individual subjects showed that propranolol attenuated the later increases in femoral blood flow and vascular conductance.

Also, some fundamental limitations related to autonomic blockades were involved in this study. First, since propranolol was injected into systemic circulation, propranolol blunted the pressor response during the later period of one‐legged exercise. The blunted perfusion pressure might account for the attenuated increases in Oxy‐Hb and femoral blood flow of the nonexercising limb at that period. However, the increase in femoral vascular conductance of the nonexercising leg was blunted by propranolol (Fig. 3). Since we noticed that spontaneous fluctuation of MAP (approximately by 5–20 mmHg) had little influence on the Oxy‐Hb (unpublished observation), it is unlikely that the Oxy‐Hb response during exercise was simply due to changes in perfusion pressure. Second, it is known that propranolol may enter into the central nervous system and affect brain function (Hoffman 2001). However, a small dose of propranolol administered in this study altered neither resting MSNA to a lower leg nor the response in MSNA during lower body negative pressure (Jacobsen et al. 1992). The RPE during exercise and vividness score during mental imagery were not influenced by propranolol. Thus, we suppose that the central effect of propranolol was the minimum in this study if any. Similarly, the dose of atropine administered in this study was as low as to have almost no detectable effect on the central nervous system (Brown and Taylor 2001), although the RPE increased slightly following subsequent atropine. Third, this study did not examine the effects of the autonomic blockades in a reverse order, because we have already obtained the sole effect of atropine on the Oxy‐Hb responses during voluntary one‐legged exercise and motor imagery (Ishii et al. 2013).

### Perspective and conclusion

It is currently thought that vasodilator mechanisms via locally released vasoactive substances and/or mechanical deformation contribute to exercise hyperemia (Saltin et al. 1998; Wray et al. 2005; Clifford 2007; Joyner and Wilkins 2007; Kirby et al. 2007). For example, a reduction in superoxide production by muscle contraction evokes increased nitric oxide in the interstitium, which in turn causes relaxation of vascular smooth muscles (Golub et al. 2014). Such local mechanisms do not account for the increase in blood flow of noncontracting muscle and a neurohumoral mechanism should play a role in the muscle hyperemia as observed during motor imagery (Ishii et al. 2013), mental stress (Dietz et al. 1994; Halliwill et al. 1997), and feeding (Mitchell et al. 2013). As possible neurohumoral factors, we propose that rapid cholinergic and delayed *β*‐adrenergic vasodilator mechanism cooperate to increase muscle blood flow not only in noncontracting but also in contracting skeletal muscle.

## Acknowledgment

We thank Ms. Maiko Takashima of GE Healthcare for her technical support.

## Conflict of Interest

None declared.
